# Lnc-ZEB2-19 Inhibits the Progression and Lenvatinib Resistance of Hepatocellular Carcinoma by Attenuating the NF-κB Signaling Pathway through the TRA2A/RSPH14 Axis

**DOI:** 10.7150/ijbs.85270

**Published:** 2023-07-16

**Authors:** Mingbo Cao, Yupeng Ren, Yuxuan Li, Junfeng Deng, Xiaorui Su, Yongchang Tang, Feng Yuan, Haixia Deng, Gaoyuan Yang, Zhiwei He, Bo Liu, Zhicheng Yao, Meihai Deng

**Affiliations:** 1 Department of Hepatobiliary Surgery, The Third Affiliated Hospital, Sun Yat-sen University, Guangzhou, 510630, China.; 2 Department of Hepatobiliary & Pancreatic Surgery, The Third Affiliated Hospital, Sun Yat-sen University, Guangzhou, 510630, China.; 3 Department of General Surgery, Qilu Hospital, Shandong University, Jinan, China.; 4 Department of Hepatobiliary Surgery, The First Affiliated Hospital of Guangzhou Medical University, Guangzhou, 510120, China.

**Keywords:** Hepatocellular Carcinoma, Lnc-ZEB2-19, TRA2A, mRNA stability, Lenvatinib resistance

## Abstract

Long non-coding RNAs have been reported to play a crucial role in tumor progression in hepatocellular carcinoma (HCC). Lnc-ZEB2-19 has been validated to be deficiently expressed in HCC. However, the capabilities and underlying mechanisms of lnc-ZEB2-19 remain uncertain. In this study, we verified that the downregulation of lnc-ZEB2-19 was prevalent in HCC and significantly correlated with the unfavorable prognosis. Further in vitro and in vivo verified that lnc-ZEB2-19 notably inhibited the proliferation, metastasis, stemness, and lenvatinib resistance (LR) of HCC cells. Mechanistically, lnc-ZEB2-19 inhibited HCC progression and LR by specifically binding to transformer 2α (TRA2A) and promoting its degradation, which resulted in the instability of RSPH14 mRNA, leading to the downregulation of Rela(p65) and p-Rela(p-p65). Furthermore, rescue assays showed that silencing RSPH14 partially restrained the effect of knockdown expression of lnc-ZEB2-19 on HCC cell metastatic ability and stemness. The findings describe a novel regulatory axis, lnc-ZEB2-19/TRA2A/RSPH14, downregulating the nuclear factor kappa B to inhibit HCC progression and LR.

## Introduction

Hepatocellular carcinoma (HCC), the most common type of liver cancer, is the third most common cause of cancer-associated deaths worldwide, with up to 80,000 deaths per year^1^. Due to its insidious pathogenesis, approximately 70-80% of patients have developed late stages upon diagnosis, requiring targeted therapy^2^-^4^. Lenvatinib is one of the multi-target tyrosine kinase inhibitors (TKIs) targeting a collection of receptors primarily affecting angiogenesis^5^, ^6^. Despite the notable survival benefits of lenvatinib in advanced HCC patients, lenvatinib resistance (LR) occurs throughout the entire process of targeted therapy and significantly compromises the prognosis of HCC patients^7^. LR is a complex pathological process involving multiple changes, such as the activation of signaling pathways, epigenetic regulation, tumor microenvironment, and cancer stem cells^8^. Therefore, the complexities of the LR mechanisms are responsible for the unsatisfactory outcomes observed in most studies. Further investigations focusing on the mechanisms of LR are urgently needed to overcome this limitation.

Non-coding RNAs are termed as lncRNAs when their length exceeds 200 nucleotides. Mounting evidence suggests that lncRNAs play critical roles in the progression of most malignancies, including HCC^9^. For example, lncRNA CEBPA-DT can promote the expression of DDR2 by binding to hnRNPC, facilitating the alteration of subcellular localization, increasing the interactions between DDR2 and β-catenin, and subsequently promoting HCC metastasis^10^. In addition, by competitively binding to the HuR protein, lncRNA ADORA2A-AS1 can inhibit the interaction between HuR and FSCN1 by downregulating the expression of FSCN1, subsequently repressing AKT signaling pathway activation, thereby restraining HCC progression^11^. Moreover, lncRNAs also affect sorafenib resistance 12, 13. However, few studies have focused on the role of lncRNAs in lenvatinib resistance in HCC.

Transformer2 (TRA2) proteins are splicing regulators that were initially found to play sex-determining roles in flies^14^. TRA2A, an isoform of TRA2, has attracted increasing attention recently. TRA2A can influence tumor progression by participating in pre-mRNA alternative splicing (AS). For example, TRA2A can alter the AS process of RSRC2 by specifically binding to the intron sequence of exon 4, promoting subtype transition from RSRC2s to RSRC2l and leading to paclitaxel resistance in breast cancer^15^. In contrast, TRA2A, as an RNA-binding protein, can also be a critical and effective participator in the development of various cancers. For example, lncRNA MALAT1 can specifically bind to TRA2A and regulate the EZH2/β-catenin pathway to facilitate the progression of esophageal carcinoma^16^. Additionally, by binding to the TRA2A promoter, HIF1α can upregulate the transcription of TRA2A, causing pancreatic cancer progression 17. According to a previous study, TRA2A was significantly upregulated in HCC^18^. However, it remains unclear whether TRA2A critically affects hepatocarcinogenesis and LR.

In the present study, we identified lnc-ZEB2-19 as a tumor suppressor gene that inhibited HCC progression and lenvatinib resistance, and its low expression was strongly associated with unfavorable outcomes. Furthermore, our study demonstrated that lnc-ZEB2-19 inhibited HCC progression and LR by specifically binding to RBP TRA2A to affect the stability of RSPH14 mRNA.

## Materials and Methods

### Clinical sample preparation and Ethics statement

Hepatocellular carcinoma and paracancerous tissues were acquired from inpatients receiving anatomical and non-anatomical hepatectomy with no chemoradiotherapy in the Department of Hepatobiliary Surgery at The Third Affiliated Hospital of Sun Yat-sen University from January 2017 to January 2019. Written informed consent was obtained from all the patients. All tissues obtained from the inpatients were diagnosed with HCC by histopathological assessment. Recurrence-free survival (RFS) and overall survival (OS) were calculated from the date of surgery until HCC recurrence or patient death. The study protocol was approved by The Ethics Committee of The Third Affiliated Hospital of Sun Yat-Sen University.

### RNA-Seq

Hep3B cells in the lnc-ZEB2-19 overexpression and control groups were used for RNA sequencing screening (BGI Genomics Co., Ltd., Shenzhen, China). The false discovery rate (FDR) was controlled by altering the *p* values measured using the Benjamini and Hochberg methods. The cutoff values were set as log2 (fold change |FC|) > 2 and FDR < 0.05 to identify differentially expressed genes.

### Cell culture

The American Type Culture Collection (ATCC, Manassas, VA, USA) supplied the HepG2, 293T, PLC/PRF/5, and Hep3B cell lines. Normal human liver cell lines L02, MIHA, QGY, MHCC-LM3, MHCC97-H, MHCC97-L, and Huh-7 were purchased from Cellcook Biotech (Guangzhou, China). We cultured the cells in Dulbecco's modified Eagle's medium (DMEM) with 5% CO_2_ at 37 °C, which contains 10% FBS and 1× streptomycin-penicillin. Cells were passaged at 80% confluence.

### Transfection of HCC cells

Hanbio Biotechnology (Shanghai, China) designed and synthesized an overexpression plasmid vector (Lv-ZEB2-19) and an empty vector (Lv-Con). Short hairpin RNA targeting lnc-ZEB2-19 (sh-lnc), TRA2A (sh-TRA2A), and RSPH14 (sh-RSPH14) were purchased from IGE Biotechnology (Guangzhou, China) to knockdown the expression of individual genes. In addition, 293 T cells were used for transfection to obtain viruses. The transfection procedure was conducted in six‐well plates until the seeded cells grew to approximately 50-70%, based on the manufacturer's instructions. The Polybrene reagent (Biosharp, Beijing, China) was used to promote the transfection efficiency, and stable cell lines were selected for puromycin (1-2 μg/ml, Biosharp, Beijing, China) resistance. Supplementary Table 3 displays all shRNA sequences.

### RNA extraction and Real-time Quantitative PCR

Total RNA was extracted from HCC tissues and cell lines using the TRIzol reagent (Vazyme, Nanjing, China). HiScript III All-in-one RT SuperMix perfect reagent (Vazyme, Nanjing, China) was used to generate cDNA. ChamQ Universal SYBR qPCR Master Mix (Vazyme, Nanjing, China) was used for qRT-PCR. The relative Ct (2^-ΔCT^) method was employed to obtain the expression level. Supplementary Table 2 displays all primer sequences.

### Subcellular fractionation location

A nucleoplasmic separation kit (IGE Biotechnology, Guangzhou, China) was used to separate the nuclear and cytosolic fractions, followed by qRT-PCR to testify lnc-ZEB2-19 expression. U6 and GAPDH were controls.

### Immunofluorescence (IF) analyses

Each well with coverslips in 24-well plates was plated with 5 × 10^4^ cells. A 24-well plate was drawn and immobilized in 4% paraformaldehyde solution for 15 min. PBS with 0.2% Triton X-100 was used for permeabilization, and the blocking solution was added to a 24-well plate for 1 h. The primary antibodies were employed to incubate the cells overnight at a 4°C environment after the blocking procedure. After washing with PBST, the cells were incubated with suitable secondary antibodies (1:500; Boster Biology, Wuhan, China). DAPI reagent (RiboBio Biotechnology, Guangzhou, China) was applied after the incubation was terminated, the coverslips were mounted on slides, and an Axio Imager 2 microscope (Zeiss, Germany) was used to obtain images.

### Fluorescence in situ hybridization (FISH)

The probes for lnc-ZEB2-19, 18S, and U6 were Cy3-labeled and synthesized by RiboBio (RiboBio Biotechnology, Guangzhou, China). An Axio Imager 2 microscope (Zeiss, Germany) was used to generate the images required for this study.

### Immunohistochemistry (IHC)

The IHC analyses were conducted according to a protocol used in a previous study^23^. Sections obtained from HCC patients were dewaxed in xylene solution and hydrated in alcohol at different concentrations. A pressure cooker containing EDTA antigen restoration solution (pH8; ZSGB-BIO, China) was used to restore the antigens. After 25min, the sections were blocked in a 3% hydrogen peroxide solution, the primary antibodies were added, and all sections were incubated at 4°C for 12-18h. The sections were subsequently stained with DAB (Dako REAL™) after incubation with the secondary antibody, and the nuclei were stained with hematoxylin after washing off the secondary antibody.

Paraffin-embedded tissues from subcutaneous xenograft tumors were stained with hematoxylin-eosin (HE) and IHC. The surgical procedures were the same as those previously described.

### Western blot assay and Antibodies

Western blotting was performed to evaluate the protein expression. Antibodies against GAPDH (1:1000, affinity), α-tubulin (1:1000, Servicebio), and β-actin (1:1000, affinity) were used as controls, and TRA2A (1:1000; Abcam) and RSPH14 (1:1000; affinity) were used for the experiments. The enhanced chemiluminescence (ECL) chromogenic substrate (Advansta, CA, USA) was applied to develop the protein bands, and images were conducted by Tanon^TM^ 5200CE Chemi-Image System (Shanghai, China).

### CCK-8 and Colony formation assays

Cell proliferation was verified using the Cell Counting Kit-8 reagent (RARbio, China). Briefly, cells (1000-2000 cells per well) were inoculated and cultured in 96-well plates. After adding 10 µL CCK-8 solution for 2 h, the cell viability of each group was evaluated. For colony formation, 500-1000 cells were inoculated into six-well plates and stained with crystal violet after two weeks. An Axio Imager 2 microscope (Zeiss, Germany) was used to generate the images.

### Wound healing and Transwell Assays

After stable cell lines were constructed, the cells were inoculated into six-well plates and routinely cultured until they reached >95% confluence. The scratches of each group were conducted using 200 µL pipette tips. The widths of the scratches were imaged using a microscope at 0 and 48 h, respectively. For the transwell assay, each group containing 1 × 10^5^ HCC cells was mixed with medium without FBS in the upper chamber (NEST, China; Corning, USA) with or without Matrigel (BD Biosciences, China), and medium containing 15% FBS was added to the lower chamber. Crystal violet (0.1%) was applied to stain the penetrating cells for 60 minutes after 24h of incubation. An inverted microscope (Olympus, Tokyo, Japan) was used to calculate the number of invaded or migrated cells.

### Sphere-formation assay

Next, 0.5% N2 and 1% B27 (Gibco, USA), bFGF and EGF (50 ng/mL, Aladdin, China), and 1% glutamine (Macklin, China) were mixed with the cells (1 × 10^3^ cells per well) and then inoculated into ultralow adhesion plates containing FBS-free DMEM/F12. Cells were cultivated in 37 °C incubation with 5% CO_2_ for 15 days.

### Measurement of cytotoxicity

Cells in a 96-well plate (5000 cells per well) were treated with or without a gradient concentration of lenvatinib. The IC50 value was calculated at 24-48 h according to the CCK-8 experimental procedure.

### Actinomycin D treatment

Cells were seeded in 6-well plates with 2 μg/mL actinomycin D (APExBIO, USA) added for blocking transcription and then digested with TP to obtain total RNA for qRT-PCR in different duration.

### RNA pull-down and RNA immunoprecipitation

An RNA pull-down kit (Bersinbio, Guangzhou, China) was used to obtain the RNA-protein complex. Western blotting was used to evaluate the target protein expression. For RNA immunoprecipitation (RIP), each group containing 2 × 10^7^ HCC cells was analyzed using an anti-TRA2A antibody (#ab138448, Abcam, UK) and an RNA immunoprecipitation kit (Bersinbio, Guangzhou, China). The enrichment of lnc-ZEB2-19 and RSPH14 was verified by qRT-PCR and gel electrophoresis.

### Xenograft in nude mice

The Guangdong Medical Laboratory Animal Center (Guangzhou, China) supplied the 4 weeks old BALB/c nude mice, which were used to establish a subcutaneous xenograft tumor model. Hep3B cells (2 × 10^6^/100 µL) from each group were inoculated into the right thigh tissue for tumor development. The length (L) and width (W) of the xenograft tumors were calculated using calipers to obtain the tumor volume (V) according to the equation (V = L × W2 × 0.524). After four weeks, the mice were sacrificed, and tumor nodules were excised to construct the tumor growth curve. Tumor tissues were fixed with 4% formaldehyde for IHC and HE staining. The experiments were approved by the Institutional Animal Ethics Committee of the Third Affiliated Hospital of Sun Yat-sen University.

### Statistical Analysis

Each experiment was conducted in triplicate. The data were analyzed by GraphPad Prism 8.0 and expressed as mean ± standard deviation. When comparing two samples, statistical significance was determined using Student's t-test or Wilcoxon test. Statistical significance was set at *p* < 0.05.

## Results

### Lnc-ZEB2-19 is downregulated in HCC tissues and correlated with patient prognosis

**Figure 1:**
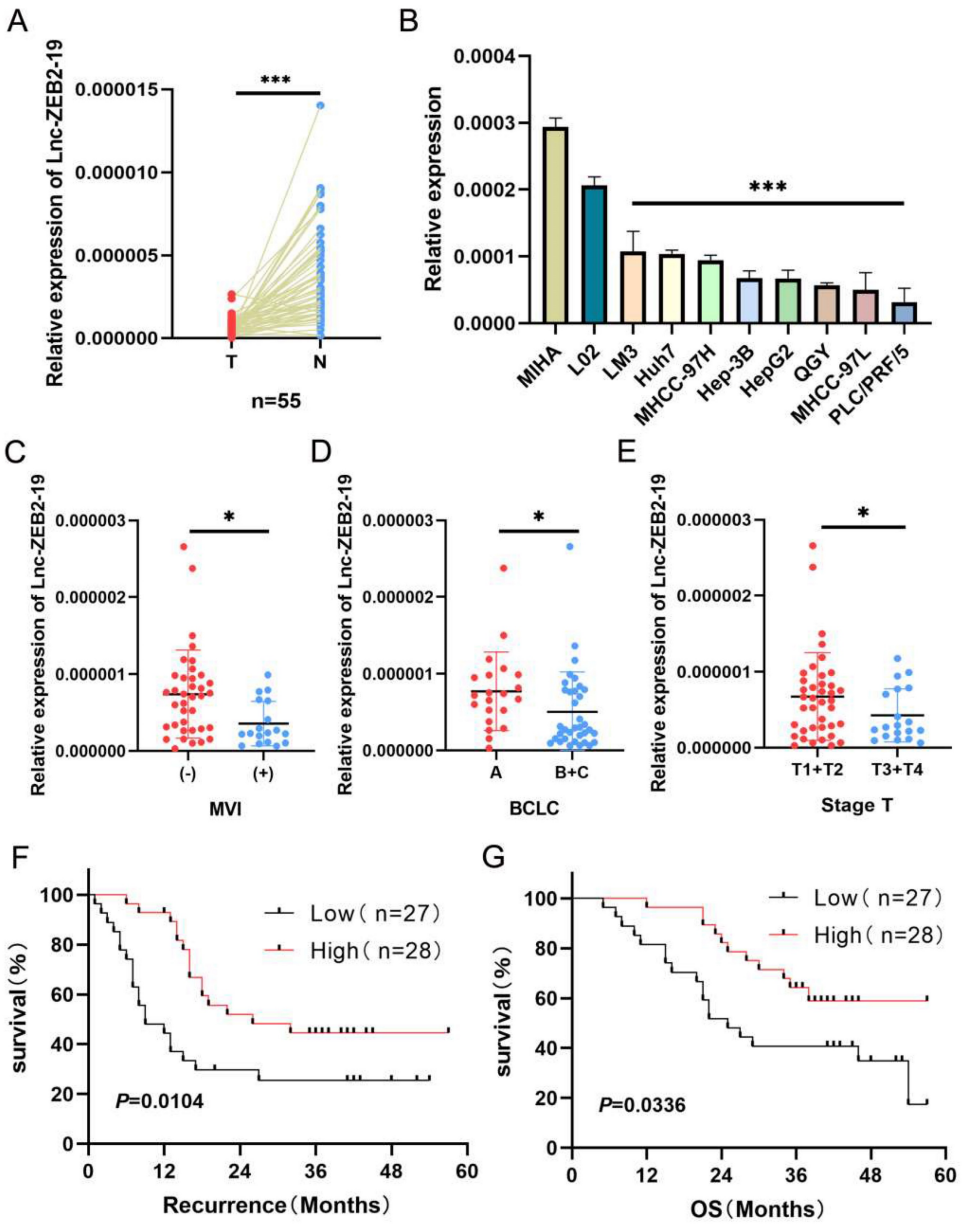
** lnc-ZEB2-19 is down-regulated in HCC tissues and correlated with patient prognosis. (A, B)** Relative lnc-ZEB2-19 expression in paired HCC tissues compared with noncancerous tissue (n = 55) and HCC cell lines.** (C, D, E)** Relative lnc-ZEB2-19 expression in subgroups. **(F, G)** Kaplan-Meier recurrence-free survival and overall survival curves according to lnc-ZEB2-19 expression levels.

At the beginning of this study, we investigated the expression status of lnc-ZEB2-19. Lnc-ZEB2-19 was extremely downregulated in hepatocellular carcinoma tissues (Fig. 1A) as well as in HCC cell lines (Fig. 1B). Subsequently, 55 patients with HCC were divided into a relatively high lnc-ZEB2-19 expression group (n=28, expression levels≥ median) and a relatively low lnc-ZEB2-19 expression group (n=27, expression levels < median) to assess the correlation between lnc-ZEB2-19 expression and malignant tumor characteristics. Lnc-ZEB2-19 expression was correlated with macrovascular invasion (MVI) (*P*=0.0227), TNM stage (*P*=0.0496), and BCLC stage (*P*=0.0261), and subgroup analysis also indicated that the expression of lnc-ZEB2-19 was relatively lower in patients with MVI (+), advanced BCLC, and TNM stage (Fig. 1C, D, E). Furthermore, the recurrence-free survival (RFS) and overall survival (OS) curves were drafted by Kaplan-Meier analysis and verified by the log-rank test, which demonstrated that HCC patients with relatively low expression of lnc-ZEB2-19 had poorer RFS and OS than those with high lnc-ZEB2-19 expression (Fig. 1F, G). Therefore, lnc-ZEB2-19 may be a useful biomarker in patients with HCC. The correlation between lnc-ZEB2-19 expression and the baseline features of clinical patients is shown in Table 1.

### Lnc-ZEB2-19 inhibits the progression and lenvatinib resistance of HCC

**Figure 2:**
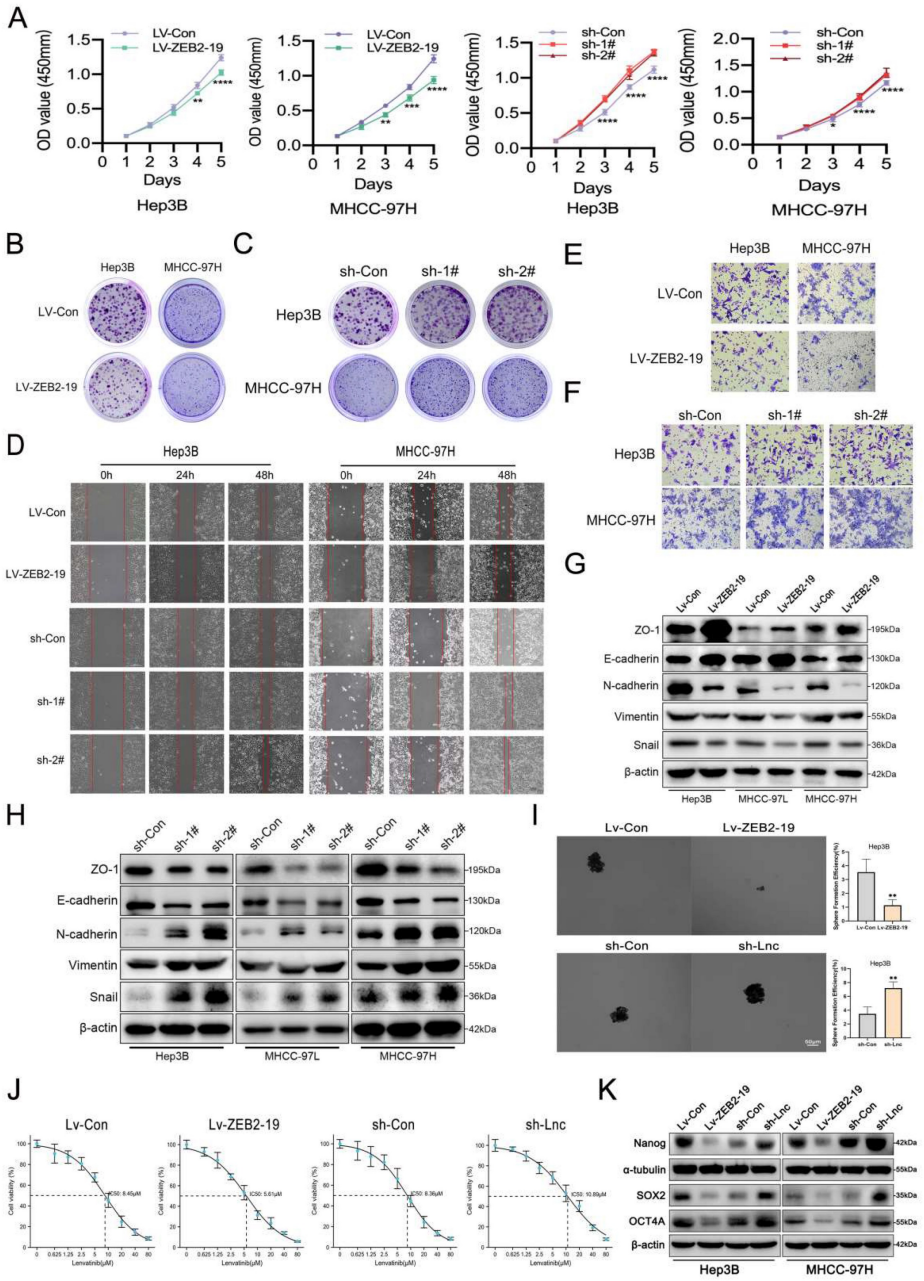
** lnc-ZEB2-19 inhibits the progression and Lenvatinib resistance of HCC cells.** In this figure, Hep3B and MHCC-97H cells overexpressing or knocking down lnc-ZEB2-19 and the control group were named Lv-ZEB2-19, Lv-Con, sh-Lnc, and sh-Con. **(A)** CCK8 assay was conducted to confirm the viability of each group of HCC cells. The effects of lnc-ZEB2-19 on HCC cell colony formation ability** (B, C)** and migration ability from wound healing assays** (D)** and invasion ability from Transwell assays **(E, F)** and sphere-formation ability **(I)**. Western blot assays were applied to verify the relative protein level of EMT **(G, H)** and stemness **(K)**. The sensibility of lenvatinib was validated through IC50 assays **(J)**.

To explore the biological function of lnc-ZEB2-19 in HCC, stable cell lines overexpressing or knocking down lnc-ZEB2-19 were established using the Hep3B, MHCC97-H, and MHCC97-L depending on the expression status of lnc-ZEB2-19 in HCC cell lines. Overexpression and knockdown efficiencies were verified by qRT-PCR (Supplementary Figure S1A, B), and sh-1# and sh-2# were selected based on their satisfactory elimination efficiencies. Ectopic expression of lnc-ZEB2-19 notably inhibits cell proliferation, whereas knockdown of lnc-ZEB2-19 increases the ability of cell proliferation and colony formation compared to control cells significantly (Fig.2A, B, C; Supplementary Figure S1C, D, E). Wound healing and transwell assays verified that overexpression of lnc-ZEB2-19 enormously decreased the healing proportion and number of penetrated cells, whereas lnc-ZEB2-19 knockdown had the opposite effect on metastatic ability in HCC Cells (Fig.2D, E, F; Supplementary Figure S1F, G). A western blot assay was employed to verify the change in the protein levels of epithelial-mesenchymal transition (EMT). The results showed that upregulating of lnc-ZEB2-19 extremely restrained the EMT of HCC cells; in contrast, silencing lnc-ZEB2-19 notably promoted the invasive ability (Fig.2G, H; Supplementary Figure S1H). Next, we explored whether lnc-ZEB2-19 could affect stemness, which critically affects the LR. Sphere-formation and IC50 assays were conducted, and lnc-ZEB2-19 overexpression significantly inhibited the sphere-formation capability and IC50 value of HCC cells, whereas lnc-ZEB2-19 knockdown notably facilitated the efficiency of sphere formation and resistance to lenvatinib (Fig.2I, J). To further demonstrate the impact of lnc-ZEB2-19 on stemness, western blotting (WB) assays were conducted to validate the variation in the expression of stemness markers, including Sox2, Nanog, and Oct4. The results revealed that stemness markers were upregulated after lnc-ZEB2-19 knockdown and were notably downregulated when lnc-ZEB2-19 was overexpressed (Fig.2K). These results suggest that lnc-ZEB2-19 can restrain proliferation, migration, invasion, stemness, and resistance to lenvatinib in HCC cells.

### Lnc-ZBE2-19 interacts with TRA2A

**Figure 3:**
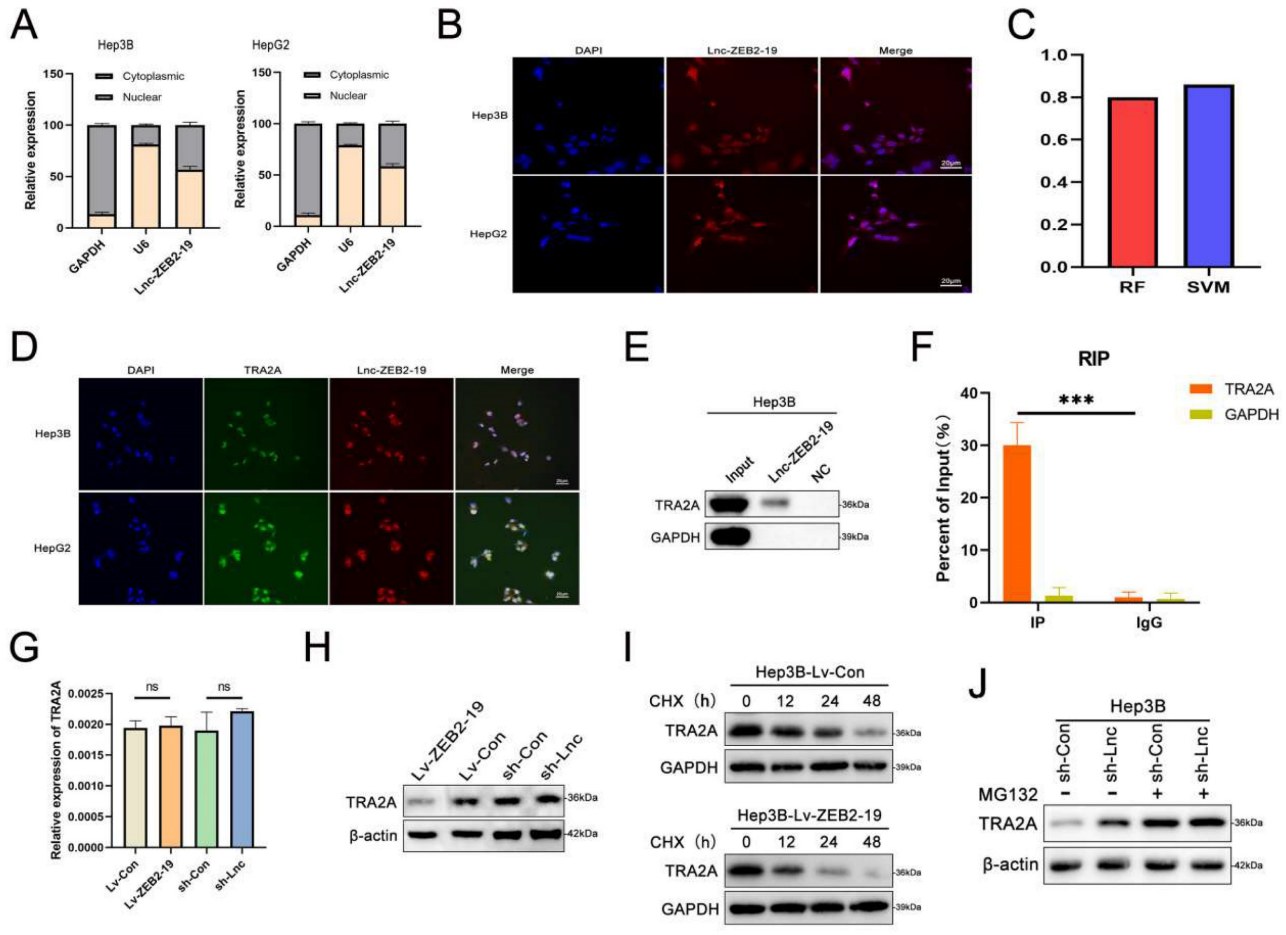
** lnc-ZEB2-19 interacts with TRA2A. (A, B)** Subcellular localization of lnc-ZEB2-19 was confirmed through subcellular fractionation assay and FISH. **(C)** The interaction between lnc-ZEB2-19 and TRA2A was strongly recommended by RPI-seq platforms. FISH-IF **(D)**, RNA pull-down **(E),** and RIP **(F)** assays were used to demonstrate the interaction of lnc-ZEB2-19 and TRA2A. **(G, H)** qRT-PCR and western blot assays showed that TRA2A was regulated by lnc-ZEB2-19 at the protein level, not the mRNA level. The regulation of lnc-ZEB2-19 to TRA2A protein was validated by cycloheximide **(I)** and MG132 **(J)**.

To further investigate the potential molecular mechanisms of lnc-ZEB2-19 in HCC, we first performed FISH and nucleoplasmic separation assays. As shown in Fig. 3A and B, Lnc-ZEB2-19 was located in the cytoplasm and nucleus, with the nucleus predominating, suggesting that lnc-ZEB2-19 may play a regulatory role primarily by interacting with RNA binding proteins (RBPs). Candidate proteins were predicted using the bioinformatics tool CatRAPID, and TRA2A was selected because it had the most significant number of RNA-binding motifs (Supplementary Figure S2A). Validation of the binding capability between lnc-ZEB2-19 and TRA2A is highlighted in the RPISeq database (Fig. 3C). RNA pull-down, RIP, and IF assays were performed to verify the predicted results. The co-localization of TRA2A and lnc-ZEB2-19 was demonstrated by the RNA FISH-IF assay (Fig. 3D). The RNA pull-down assay showed that the lnc-ZEB2-19 probe, not its anti-sense probe, significantly pulled down the TRA2A protein (Fig. 3E). RIP and gel electrophoresis assays further verified that TRA2A could specifically precipitate lnc-ZEB2-19 (Fig. 3F; Supplementary Figure S2B). Considering the growing evidence that lncRNAs may regulate their binding targets^19^, qRT-PCR and WB assays were conducted to demonstrate whether lnc-ZEB2-19 affected TRA2A expression. The results revealed that lnc-ZEB2-19 negatively regulates TRA2A but not TRA2A mRNA (Fig. 3G, H). Furthermore, a cycloheximide chase assay was performed to explore how lnc-ZEB2-19 decreased TRA2A protein expression. As shown in Fig. 3I, the half-life of the TRA2A protein was markedly reduced in the overexpression lnc-ZEB2-19 group compared to that in the control group. Moreover, the upregulation of TRA2A induced by knocking down lnc-ZEB2-19 expression could be reversed by treatment with MG132 (Fig. 3J), indicating that lnc-ZEB2-19 interacts with TRA2A and accelerates its protein degradation.

### TRA2A acts as an oncogene in HCC progression

**Figure 4:**
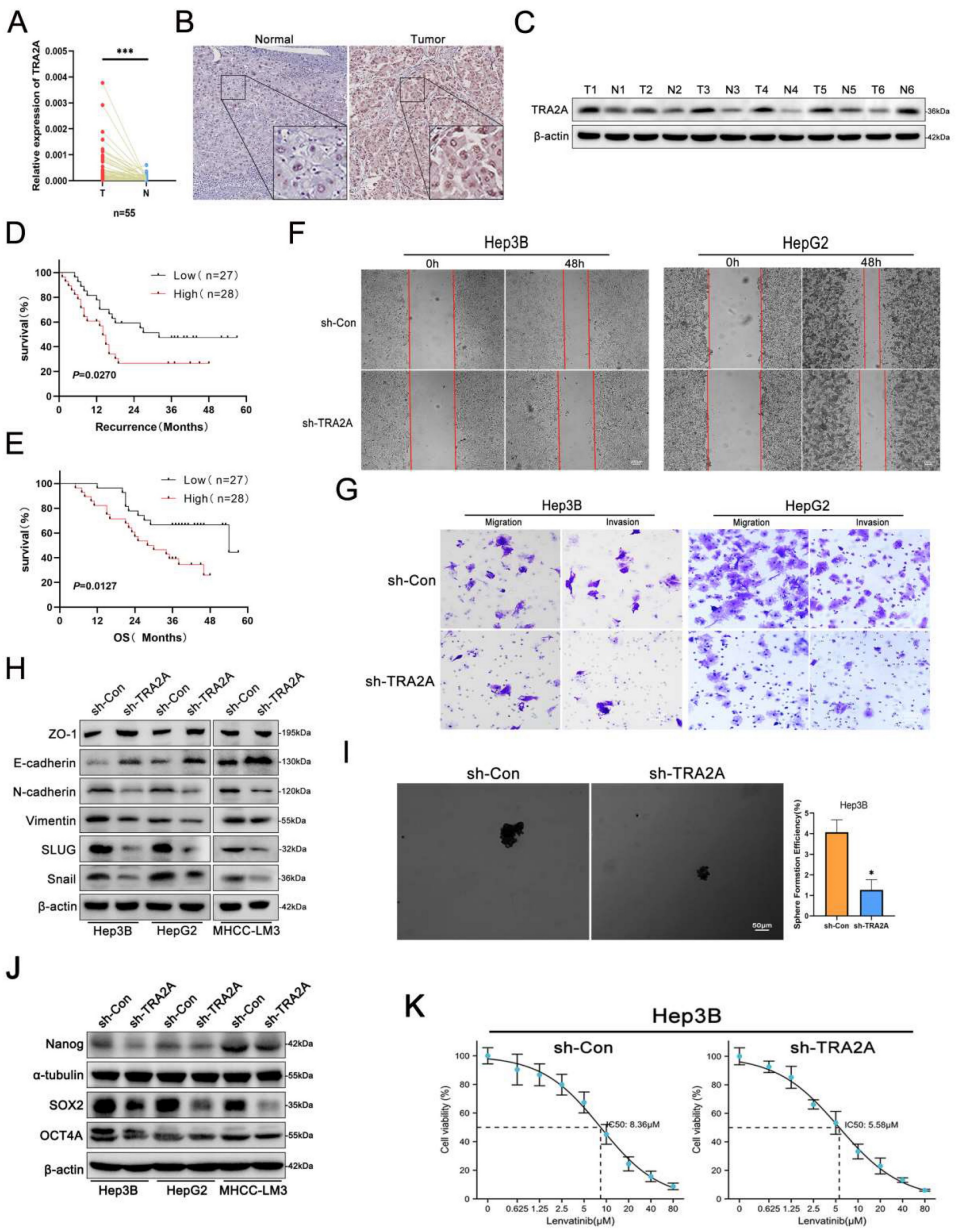
** TRA2A acts as an oncogene in HCC cell progression**. The expression of TRA2A was verified by qRT-PCR **(A)**, immunohistochemistry **(B)**, and western blot **(C)**. (**D**) The Kaplan-Meier plot between the expression level of TRA2A and RFS in patients with HCC (*P* = 0.0279). **(E)** The Kaplan-Meier plot between the expression level of TRA2A and OS in patients with HCC (*P* = 0.0127). The effects of TRA2A on HCC cell migration ability from wound healing assays** (F)** and invasion ability from Transwell assays **(G),** and sphere-formation ability **(I)**. Western blot assays demonstrated the variation of EMT **(H)** and stemness **(J)** protein levels. The sensibility of lenvatinib was confirmed by IC50 assays **(K)**.

Considering that lnc-ZEB2-19 regulates TRA2A protein expression, we investigated the role of TRA2A in HCC metastasis and LR. The online GEPIA Expression Profiling Interactive Analysis database showed that TRA2A was highly upregulated in HCC tissues (Supplementary Figure S2C). Identical results were obtained by qRT-PCR (Fig. 4A), immunohistochemistry (Fig. 4B), and western blotting (Fig. 4C). Additionally, a negative correlation was observed between TRA2A expression and RFS and OS of patients with HCC in our data as well as in the GEPIA database (Fig. 4D, E; Supplementary Figure S2D, E). Next, the upregulation of TRA2A in HCC cell lines was validated (Supplementary Figure S2F), and sh-1# was selected for subsequent functional experiments after determining the transfection efficiency (Supplementary Figure S2G, H).

HCC cell viability was significantly reduced after TRA2A silencing (Supplementary Figure S2I, J). Furthermore, in the wound healing and transwell assays, the proportion of cells climbing the distance and the number of penetrated cells in the TRA2A-knockdown group were much lower than those in the control group (Fig. 4F, G; Supplementary Figure S2K, L). Western blotting also revealed that epithelial-mesenchymal transition (EMT) was significantly attenuated after silencing TRA2A expression (Fig. 4H). Moreover, western blot assays were conducted to further verify the effect of TRA2A knockdown on cell stemness, sphere formation, and IC50. TRA2A knockdown distinctly restrained not only stemness as well as lenvatinib resistance in HCC cells (Fig. 4I, J, K). These findings indicate that downregulation of TRA2A can significantly inhibit HCC cell metastasis, stemness, and resistance to lenvatinib, corresponding with the suppressive role of overexpressing lnc-ZEB2-19.

### Lnc-ZEB2-19 affects RSPH14 mRNA stability by interacting with TRA2A

**Figure 5:**
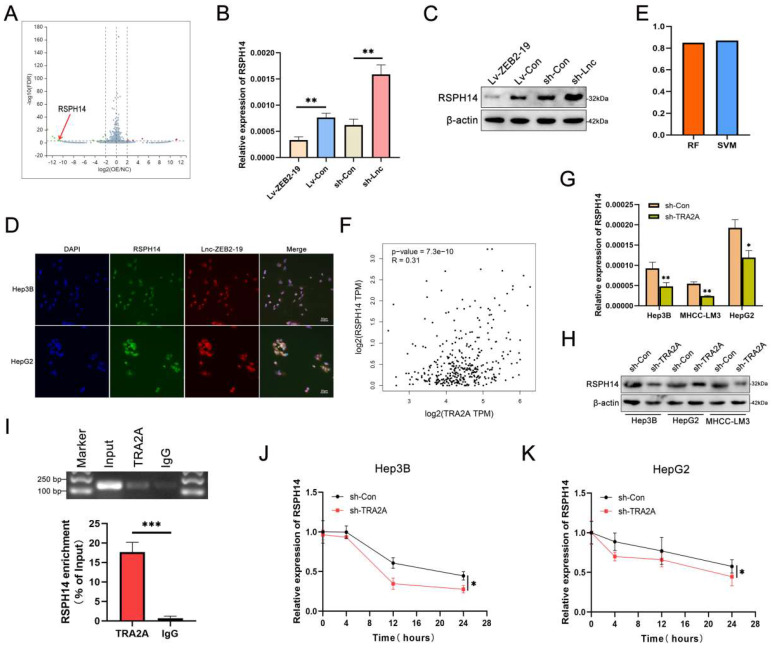
** lnc-ZEB2-19 affects RSPH14 mRNA stability by binding to TRA2A**. **(A)** The volcano map of differentially expressed genes. The regulatory role of lnc-ZEB2-19 to RSPH14 was confirmed by qRT-PCR **(B)** and western blot **(C)**. **(D)** FISH-IF assay demonstrated the colocalization of lnc-ZEB2-19 and RSPH14. The binding possibility and correlation between TRA2A and RSPH14 were recommended by the RPI-seq platform **(E)** and GEPIA database **(F)**. QRT-PCR **(G)** and western blot **(H)** assays showed the regulatory role of TRA2A to RSPH14. **(I)** RIP and gel electrophoresis assays verified the interaction between TRA2A protein and RSPH14 mRNA. **(J, K)** The effects of TRA2A on RSPH14 mRNA stability were validated by actinomycin D.

To identify the downstream genes regulated by lnc-ZEB2-19, we conducted RNA-seq in Hep3B cells overexpressing lnc-ZEB2-19 or not and plotted a volcano map (Fig. 5A). The features of the differential genes are listed in Supplementary Table 1. For matching with RNA transcriptome sequencing, the downregulated genes were singled out and validated in HCC tissues (Supplementary Figure S3A). We observed that RSPH14 had the most significant expression variance. qRT-PCR and western blot assays illustrated that RSPH14 protein and mRNA levels were regulated by lnc-ZEB2-19 (Fig. 5B, C), and the FISH-IF assay also indicated that RSPH14 shared subcellular localization with lnc-ZEB2-19 (Fig. 5D). Next, we explored the relationship between TRA2A and RSPH14 expression.

The RPISeq platform suggested the possibility of an interaction between TRA2A and RSPH14 mRNA (Fig. 5E), and a positive correlation was observed between them in the GEPIA database (Fig. 5F). qRT-PCR and western blot assays revealed that TRA2A regulated RSPH14 at both the mRNA and protein levels (Fig. 5G, H). Furthermore, RIP and gel electrophoresis assays were conducted and showed that RSPH14 mRNA was specifically precipitated by TRA2A (Fig. 5I). Considering that RBPs can affect the mRNA stability of target genes 19, we treated Hep3B cells with actinomycin D to validate whether RSPH14 mRNA stability was affected by TRA2A. As shown in Fig. 5J and K, downregulation of TRA2A markedly reduced the half-life of RSPH14 mRNA in Hep3B and HepG2 cells. These results suggest that lnc-ZEB2-19 can modulate RSPH14 mRNA expression by binding to TRA2A.

### RSPH14 is involved in the oncogenic role of lnc-ZEB2-19 in vitro

**Figure 6:**
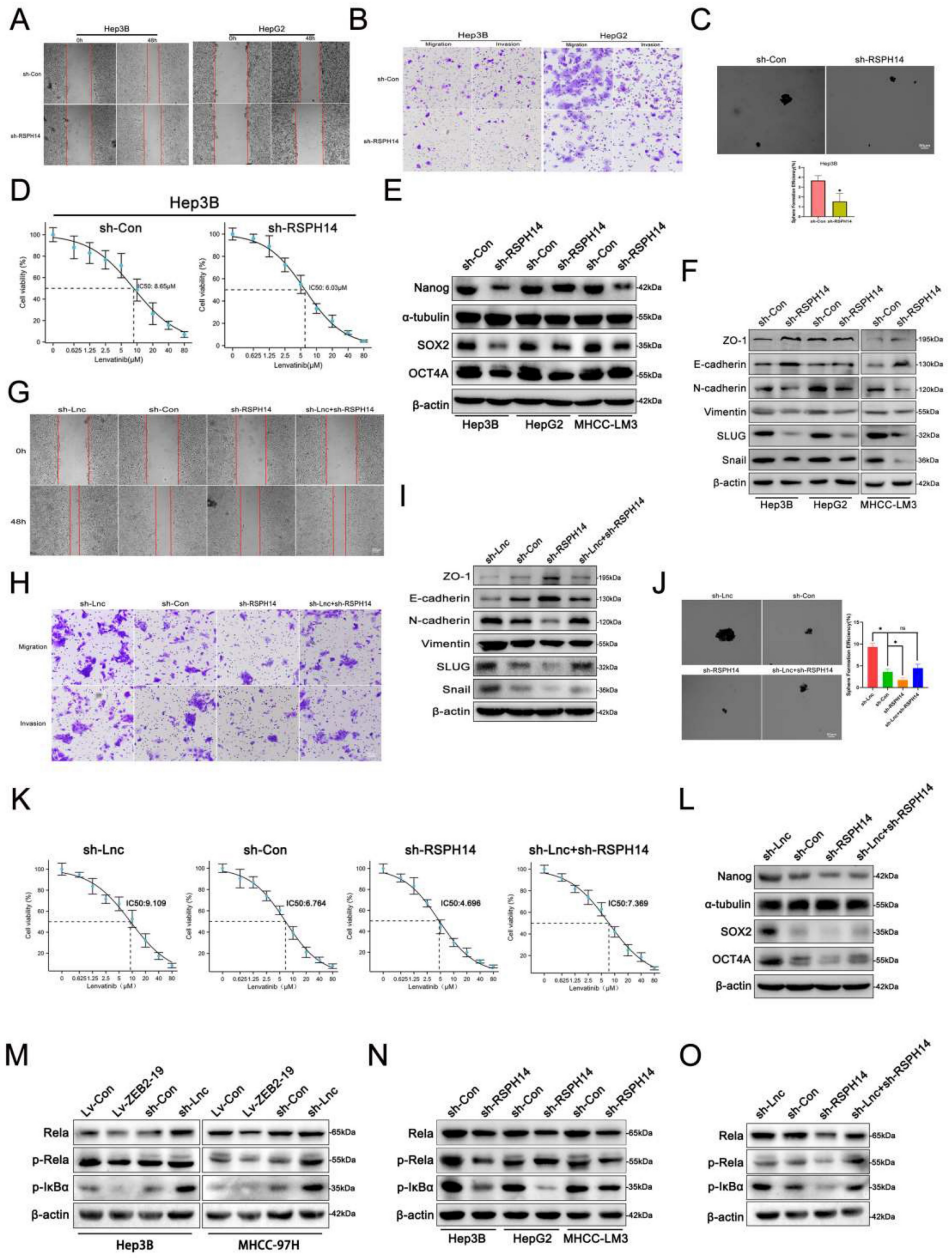
** RSPH14 is involved in the oncogenic role of lnc-ZEB2-19**. The effects of RSPH14 on HCC cell migration ability from wound healing assays** (A)** and invasion ability from Transwell assays **(B),** and sphere-formation ability **(C)**. The variation of EMT **(F)** and stemness **(E)** protein level were proved by Western blot. The sensibility of lenvatinib was verified by IC50 assays **(D)**. The rescue experiments were conducted to confirm the reverse ability to knock down RSPH14 on the migration ability **(G)**, invasion ability **(H)**, EMT **(I)**, sphere-formation ability **(J)**, stemness **(L)**, and sensibility of Lenvatinib **(K)** in HCC cells. The regulatory role of Lnc-ZEB2-19 **(M)** and RSPH14 **(N)** on the NF-κB signaling pathway was confirmed by western blot. **(O)** knocking down RSPH14 reversed the activation of the NF-κB signaling pathway induced by silencing Lnc-ZEB2-19.

Both the online GEPIA database and our data suggested that RSPH14 was observably up-expressed in HCC (Supplementary Figure S3B, C, D, E), as well as in HCC cell lines (Supplementary Figure S3F). To explore whether RSPH14 is involved in the lnc-ZEB2-19-induced inhibition of HCC progression and LR, we first performed functional assays to knock down RSPH14 after sh-3# was selected for its satisfactory inhibition efficiency (Supplementary Figure S3G, H). Cell proliferation was repressed when RSPH14 was knocked down (Supplementary Figure S4A, B). Furthermore, data from the metastasis assay showed that the knockdown of RSPH14 significantly suppressed the metastatic capability of HCC cells (Fig. 6A, B; Supplementary Figure S4C, D), as well as stemness and resistance to lenvatinib (Fig. 6C, D). This effect was also observed at the protein level (Fig. 6E, F). Rescue experiments were also conducted. The results showed that cell proliferation was promoted by downregulating lnc-ZEB2-19 and suppressed when RSPH14 was knocked down (Supplementary Figure S4E, F). Similarly, the transwell and wound healing assays results showed that the knockdown of RSPH14 notably reversed the facilitation of Hep3B cell metastasis induced by lnc-ZEB2-19 silencing (Fig. 6G, H, I; Supplementary Figure S4G, H). Moreover, RSPH14 silencing partially offset the positive effects of knocking down lnc-ZEB2-19 on stemness and resistance to lenvatinib of Hep3B cells (Fig. 6J, K, L).

RSPH14 directly regulates the expression of p65 (Rela), a critical member of the NF-κB signaling pathway^20^. Western blot assays demonstrated that not only lnc-ZEB2-19 but RSPH14 could regulate the expression of p65, p-p65, and p-IkBα with contradictory effects (Fig. 6M, N). Furthermore, reduced expression of RSPH14 functionally rescued the decrease in p65, p-p65, and p-IkBα expression after lnc-ZEB2-19 silencing (Fig. 6O). In summary, our study emphasizes the essential role of the lnc-ZEB2-19/TRA2A/RSPH14 signaling axis in the suppression of HCC metastasis and lenvatinib resistance.

### Lnc-ZBE2-19 promotes HCC cell tumorigenesis in vivo

**Figure 7:**
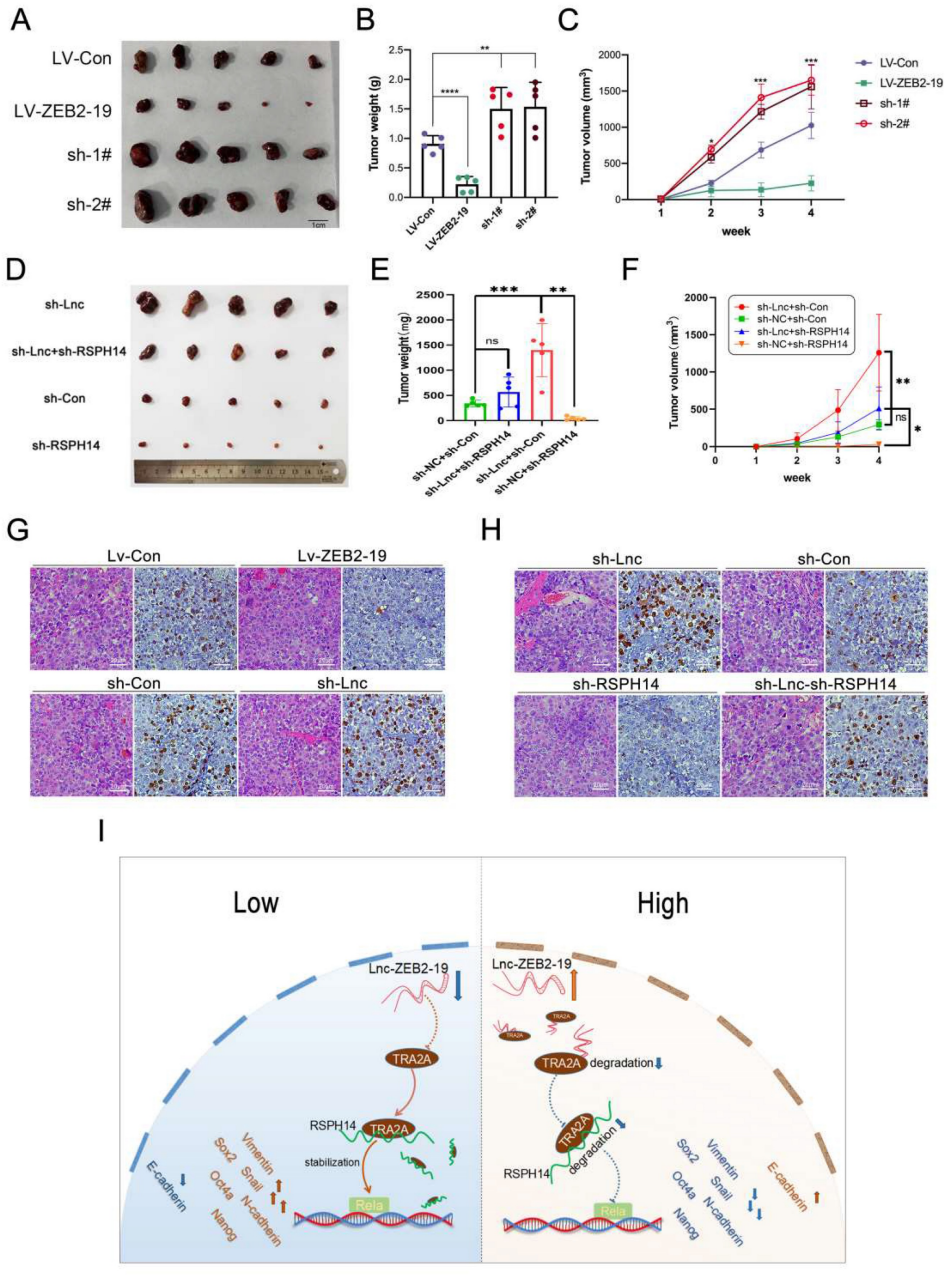
** Lnc-ZBE2-19 promotes HCC cell tumourigenesis in vivo**. Xenograft tumors from BALB/c nude mice **(A, D)**, and corresponding graphs showing the weight **(B, E)** and volume **(C, F)** of xenograft tumors from each group (n=5) on day 28. IHC staining for Ki-67 in histologic sections of xenograft tumors **(G, H)**. *P < 0.05, **P < 0.01. The data are listed as the mean ± SD.

To clarify whether lnc-ZEB2-19 could affect tumorigenesis in vivo, Hep3B cells stably overexpressing or knocked down lnc-ZEB2-19, and the corresponding control group Hep3B cells were subdermally inoculated into nude mice. Xenograft tumors were photographed (Fig. 7A), and the tumor volume and weight in the Lv-ZEB2-19 group were notably lower than those in the Lv-Con group. In contrast, the tumors in the sh-lnc group were larger and heavier than those in the sh-Con group (Fig. 7B, C). The rescue experiments were performed in vivo. Hep3B cells co-transfected with lnc-ZEB2-19 and RSPH14 shRNA were inoculated into nude mice. The results showed that silencing of RSPH14 partially reversed the enhanced effect of tumor growth induced by knocking down lnc-ZEB2-19 (Fig. 7D, E, F). Furthermore, the expression level of ki-67 in tumor sections was validated using immunohistochemical (IHC) analysis. As shown in Fig. 7G, the expression of ki-67 in the sh-lnc group was higher than that in the control group, whereas the results observed in the Lv-ZEB2-19 group were contradictory. Similarly, the enhancement of Ki-67 expression induced by lnc-ZEB2-19 knockdown was partially reversed by silencing of RSPH14 (Fig. 7H).

## Discussion

Recent evidence has shown that lncRNAs play an essential role in the regulation of HCC progression and resistance to targeted therapy 21, 22. For example, lnc-MT1JP, which is upregulated in HCC cell lines that develop LR, competitively inhibits microRNA-3-1p as a molecular sponge and thus upregulates BCL2l2 to inhibit apoptosis, ultimately causing LR^23^. Knockdown of MKLN1-AS promotes apoptosis in HCC cell lines, thus increasing their sensitivity to lenvatinib^24^. In addition, lncXIST promotes LR by interacting with EZH2 to activate the NOD2-ERK axis in HCC cells^25^. By interacting with YBX1, lncRNA USP2-AS1 increases the expression level of HIF1α to decrease the therapeutic effect of lenvatinib^26^. Moreover, lncRNA AC026401.3 can specifically bind to OCT1 and subsequently act as a scaffold to enhance its binding with OCT1 and recruit OCT1 to the E2F2 promoter, ultimately activating the transcription of E2F2 to enhance LR in HCC^27^. The above studies indicate that lncRNAs are potential new targets for LR in HCC. Therefore, finding more lncRNAs that correlate with HCC progression and researching the mechanisms underlying LR in HCC are important for achieving better survival benefits for patients with HCC.

In this study, we focused on lnc-ZEB2-19 and verified the extreme downregulation of lnc-ZEB2-19 in HCC tissues, which was closely correlated with the MVI-positive rate, advanced stage, and unfavorable outcomes in patients with HCC. Further gain- or loss-of-function assays revealed that lnc-ZEB2-19, as a tumor suppressor gene, significantly inhibited metastasis, stemness, and resistance to lenvatinib of HCC cells. Moreover, in vivo assays demonstrated that ectopic expression of lnc-ZEB2-19 restrained the proliferative capability of HCC cells while silencing lnc-ZEB2-19 significantly promoting HCC cell proliferation. These results strongly indicate that lnc-ZEB2-19 can be a robust biomarker for diagnosing and prognoses of patients with HCC and a sensitive biomarker for lenvatinib therapy.

Several studies have demonstrated that binding to RNA-binding proteins (RBPs) is one of the most important mechanisms by which lncRNAs exert their functions. For example, our previous study showed that lncRNA MALAT1 enhanced the proliferative capability of gallbladder cancer cells by epigenetically recruiting the enhancer of EZH2 to the ABI3BP promoter region^28^. By binding to PARP1, lncRNA DDX11-AS1 can effectively eliminate the interaction between PARP1 and p53, thus downregulating p53^29^.

HNRNPL and PTBP1 can also be competitively bound by the lncRNA SNHG6, thereby restraining its stabilizing effect on SETD7 and LZTFL1, causing rapid degradation of SETD7 and LZTFL1 mRNAs and eventually promoting HCC progression^30^. In addition, cancer testis lnc-CTHCC can act as a molecular scaffold to recruit hnRNP K to the promoter of YAP1, thus promoting HCC carcinogenesis and progression by activating YAP1 transcription^31^. In addition, lncRNAs can regulate protein binding by enhancing the ubiquitination or phosphorylation of target proteins. For example, LINC01554 can maintain a high expression of G3BP2 by reducing the degradation of G3BP2 induced by ubiquitination, stabilizing HDGF mRNA binding to G3BP2, and the progression of ESCC^32^. In HCC, the stability of TRIM37 and CDC27 mRNAs can be reduced because of the enhancement of PABPC4 ubiquitination induced by its combination with lncRNA RP11-286H15.1, thus repressing HCC progression^33^.

This study used catRAPID and RPISeq platforms to uncover the latent RBPs interacting with lnc-ZEB2-19. The results indicated that TRA2A might be a potential candidate protein for binding to lnc-ZEB2-19, owing to the presence of most RNA-binding motifs. The binding relationship between lnc-ZEB2-19 and TRA2A was further verified by RNA pull-down, RIP, and FISH-IF assays. Further experiments demonstrated that lnc-ZEB2-19 could regulate TRA2A protein expression but not mRNA expression. Moreover, by applying CHX and MG132 to the Hep3B cells of each group, we found that lnc-ZEB2-19 could accelerate the degradation of TRA2A by binding to its protein. Next, RNA-Seq was conducted to explore the downstream gene of lnc-ZEB2-19, and RSPH14, a gene recognized as a prognostic marker in HCC^20^ and NSCLC^34^ for the promotion of cancer progression, was selected and demonstrated to be a common target of lnc-ZEB2-19 and TRA2A. The RNA stability experiment showed that RSPH14 mRNA was rapidly degraded after TRA2A knockdown, indicating that lnc-ZEB2-19 regulates RSPH14 expression by binding to TRA2A and facilitates protein degradation, thus abrogating the protection of TRA2A protein to RSPH14 mRNA.

To better recognize the essential role of the lnc-ZEB2-19/TRA2A/RSPH14 signaling axis in restraining HCC progression and LR, a series of functional experiments were performed to investigate whether TRA2A and RSPH14 have an apparent effect on HCC cells. The results, consistent with the lnc-ZEB2-19 overexpression, showed that the knockdown of TRA2A and RSPH14 could distinctly inhibit proliferation, migration, invasion, stemness, and resistance to lenvatinib of HCC cell lines. In addition, rescue experiments demonstrated that RSPH14 knockdown effectively restored the promotion effect of lnc-ZEB2-19 silencing on HCC progression and LR. Moreover, the rescue ability of RSPH14 for silencing lnc-ZEB2-19 in vivo was highly consistent with that observed in vitro.

Extensive studies have shown that NF-kappa B, the pro-inflammatory transcription factor, exerts an essential regulation in EMT^35^ and cancer stem cells(CSCs)^36^, which were dedicated to the resistance of targeted therapy. NF-κB-p65(Rela) was one of the five subunits activating the NF-κB pathway to regulate downstream gene expression^37^. Recently, lncRNAs have been reported to interact with NF-κB-p65 to affect the activation of NF-kappa B directly or indirectly^38^. According to a previous study showing that RSPH14 could regulate Rela(p65) expression^20^, we hypothesized and confirmed that both overexpression of lnc-ZEB2-19 and knockdown of RSPH14 decreased p65, p-p65, and p-IkBα expression, whereas silencing lnc-ZEB2-19 had the opposite effect. Moreover, the rescue experiment of western blotting indicated that RSPH14 knockdown partially reversed the promotion of p65, p-p65, and p-IkBα expression induced by silencing lnc-ZEB2-19. In summary, lnc-ZEB2-19 can exert its regulatory function to p65 and p-p65 via the TRA2A/RSPH14 axis.

In conclusion, our findings proved that the downregulation of lnc-ZEB2-19 was associated with the MVI-positive rate and advanced stages and promoted HCC progression and LR. Mechanistically, lnc-ZEB2-19, as a tumor suppressor gene, inhibits the activation of NF-kappa B by interacting with TRA2A to decrease the stabilization of RSPH14 mRNA. Our results delineated a novel molecular mechanism that may provide new insights into how lncRNAs regulate mRNA and protein stability, subsequently influencing HCC progression and resistance to lenvatinib treatment. In summary, lnc-ZEB2-19 and RSPH14 can be biological targets for overcoming LR in HCC. However, Limitations are indeed inevitable. Despite the high RSPH14 expression in hepatocellular carcinoma, we observed that the relationship between the expression levels of RSPH14 and RFS and OS in HCC patients rendered opposing results to those in the GEPIA database, potentially owing to the relatively small sample size. In addition, there is a lack of lenvatinib-resistant HCC samples, which are difficult to obtain, to validate the Lnc-ZEB2-19/TRA2A/RSPH14 axis. Therefore, enlarging the sample size of lenvatinib-resistant HCC tissues is a main focus for our future research.

## Supplementary Material

Supplementary figures and tables.

## Figures and Tables

**Table 1:** Clinicopathologic correlation of lnc-ZEB2-19 expression in HCC

		Lnc-ZEB2-19 expression		*P* value
Features	Total (n=55)	Low (<median)	High (≥median)	
**Age (years)**				0.3506
<60	34	16	18	
≥60	21	11	10	
**Gender**				0.4812
Male	49	24	25	
Female	6	3	3	
**HBV infection**				0.3241
No	7	4	3	
Yes	48	23	25	
**ALB**				0.2625
<35g/L	10	4	6	
≥35g/L	45	23	22	
**AFP**				0.3514
<400μg/L	36	17	19	
≥400μg/L	19	10	9	
**HBV-DNA**				0.3491
≥10^5^	23	15	17	
<10^5^	32	12	11	
**MVI**				**0.0227**
(-)	37	14	23	
(+)	18	13	5	
**Tumor size(cm)**				0.2716
<5cm	23	10	13	
≥5cm	32	17	15	
**Tumor staging**				**0.0496**
T1+T2	36	14	22	
T3+T4	19	13	6	
**BCLC staging**				**0.0261**
A	20	6	15	
B+C	35	21	13	

## Abbreviations

HCC: hepatocellular carcinoma
LR: Lenvatinib resistance
MVI: macrovascular invasion
lncRNA: Long non-coding RNA
AS: alternative splicing
ceRNA: competing endogenous RNA
TKI: Tyrosine kinase inhibitors
TRA2A: transformer 2 alpha homolog
RSPH14: Rhabdoid tumor deletion region protein
Rela: Nuclear factor of kappa light polypeptide gene enhancer
NF-κB: nuclear factor kappa-B
EMT: Epithelial-Mesenchymal Transition
CSCs: Cancer stem cells
IHC: immunohistochemistry
qRT-PCR: quantitative real-time polymerase chain reaction
TCGA: the cancer genome atlas
OS: overall survival
RFS: recurrence-free survival
