# Supplementary material for: Lnc-ZEB2-19 Inhibits the Progression and Lenvatinib Resistance of Hepatocellular Carcinoma by Attenuating the NF-κB Signaling Pathway through the TRA2A/RSPH14 Axis
Source: Int J Biol Sci. 2023 Jul 16;19(12):3678–93. doi: 10.7150/ijbs.85270 (PMC10411463; doi:10.7150/ijbs.85270)

Supplementary Table 1:Part of differentially expressed mRNAs in Hep3B cells transfected with Lv-ZEB2-19 determined by RNA transcriptome sequencing

Gene ID	Gene Symbol	Type	log2 (OE / NC)	FDR (OE / NC)
100533467	BIVM-ERCC5	mRNA	-12.90595104	7.20E-22
100529097	RPL36A-HNRNPH2	mRNA	-12.8255551	1.10E-20
720	C4A	mRNA	-11.90839262	1.33E-10
100293534	C4B_2	mRNA	-11.57506646	3.12E-08
110354863	ZNF660-ZNF197	mRNA	-11.5028318	6.01E-08
100526832	PHOSPHO2-KLHL23	mRNA	-10.84235034	7.79E-05
2556	GABRA3	mRNA	-10.64385619	2.68E-04
116255	MOGAT1	mRNA	-10.45121111	9.15E-04
27156	RSPH14	mRNA	-10.45121111	9.13E-04
54474	KRT20	mRNA	-4.392317423	1.03E-04
100302652	GPR75-ASB3	mRNA	-4.286771649	4.95E-04
6450	SH3BGR	mRNA	-2.963736456	2.96E-04
6920	TCEA3	mRNA	-2.700439718	4.55E-04
4105	MAGEA6	mRNA	-2.460440144	8.41E-10
100529063	BCL2L2-PABPN1	mRNA	-2.08277018	4.81E-14

Supplementary Table 2: Sequences of primers used in RT-qPCR assay.

Target	Sequence (5'-3')
Lnc-ZEB2-19	Forward: CCGAAAGGAGGAGAAGCA
	Reverse: TGGCAGAAGGTGAGGGTG
RSPH14	Forward: TGCCACCCAGATTACCACTG
	Reverse: AGGTTCTCCATACAGCCTATGT
TRA2A	Forward: AGGTCGAGGTCAAGGAGACAT
	Reverse： CGCCGGTATTCTGGTGTATATG
CDH1	Forward: CGAGAGCTACACGTTCACGG
	Reverse: GGGTGTCGAGGGAAAAATAGG
CDH2	Forward: AGCCAACCTTAACTGAGGAGT
	Reverse: GGCAAGTTGATTGGAGGGATG
VIM	Forward: AGTCCACTGAGTACCGGAGAC
	Reverse: CATTTCACGCATCTGGCGTTC
SNAIL1	Forward: TCGGAAGCCTAACTACAGCGA
	Reverse: AGATGAGCATTGGCAGCGAG
ZO-1	Forward: CAGCTTGATACCTGTGAATGGG
	Reverse: TATCTGTGGTCGTGTGGGACT
GABRA3	Forward: TTACACCTTTGCATACAGAACCC
	Reverse: TTTACGATTACACCCAGACTGC
SH3BGR	Forward: TGTGACAAGGAATATGTGAGCC
	Reverse: TGAGCCCTCAGATTTGACCTG
KRT20	Forward: CTCGCTTCGGCAGCACA
	Reverse: AACGCTTCACGAATTTGCGT
TCEA3	Forward: CAAGAGGCGCAAACAAGCC
	Reverse: GGTTGGCAATACCGTCATCC
MAGEA6	Forward: TCGGAAGCCTAACTACAGCGA
	Reverse: AGATGAGCATTGGCAGCGAG
GAPDH	Forward: GGAGCGAGATCCCTCCAAAAT
	Reverse: GGCTGTTGTCATACTTCTCATGG

Supplementary Table 3: Sequences of shRNAs used in this study

Gene	sequence
sh-Con	TCCGAACGTGTCACGTA
Sh-Lnc-ZEB2-19-1#	ACCAATGGGCTCAAGAATAAA
Sh-Lnc-ZEB2-19-2#	ATCACAATAGGCCACGAATTT
Sh-Lnc-ZEB2-19-3#	AGAAGGTCAAGGTGGTCTATG
Sh-TRA2A-1#	GCTGGATGGTAGAAGAATTCG
Sh-TRA2A-2#	GGGCAAATCCAGATCCCAACA
Sh-TRA2A-3#	CGTCGAGATTCTTACTATGAT
Sh-RSPH14-1#	GCAACAGTATGGTGCGCATAA
Sh-RSPH14-2#	GCCATGAACATAGGCTGTATG
Sh-RSPH14-3#	GCTCCTTAATGTCAGCATAT

Supplementary figure S1

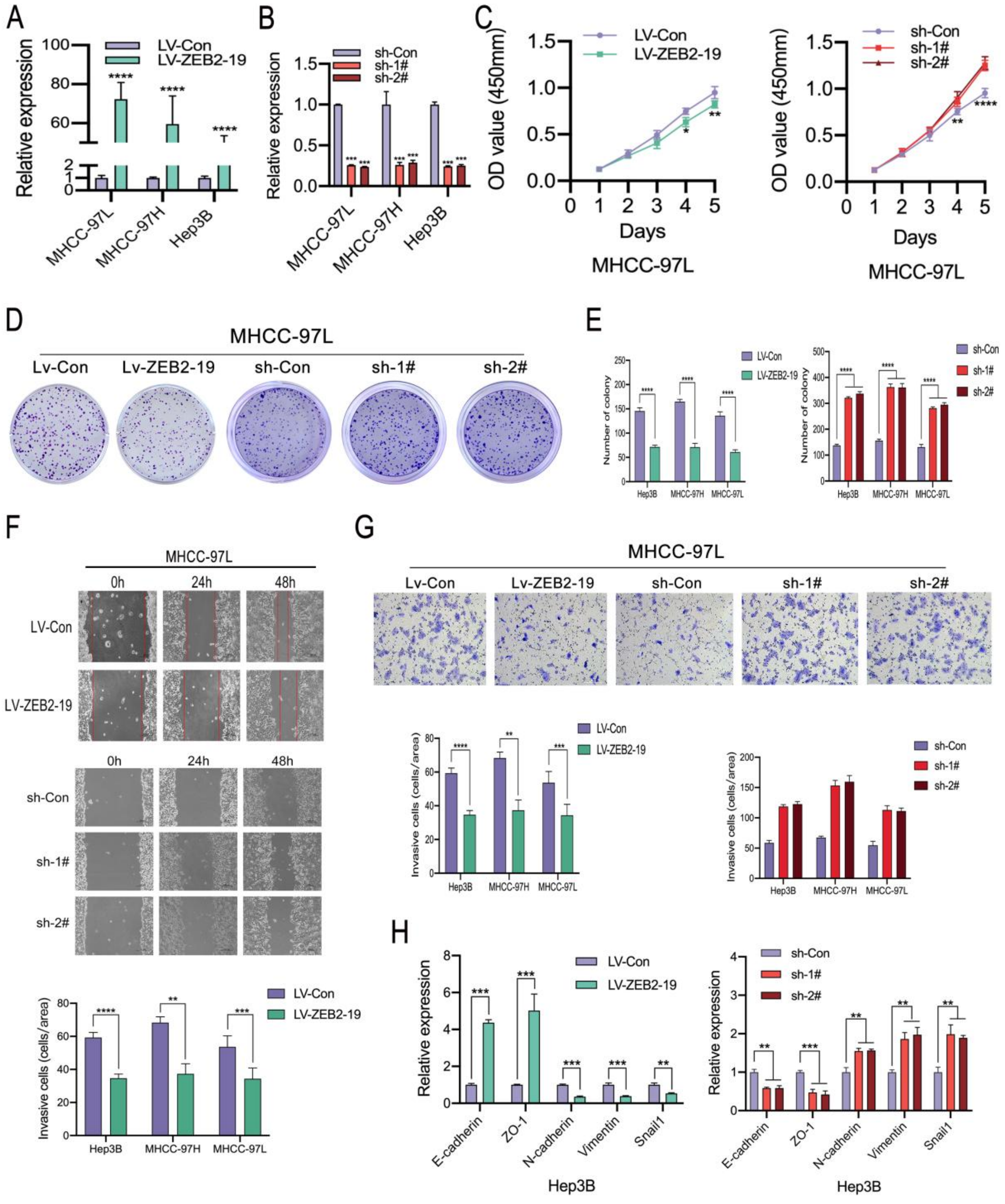

Supplementary figure S2

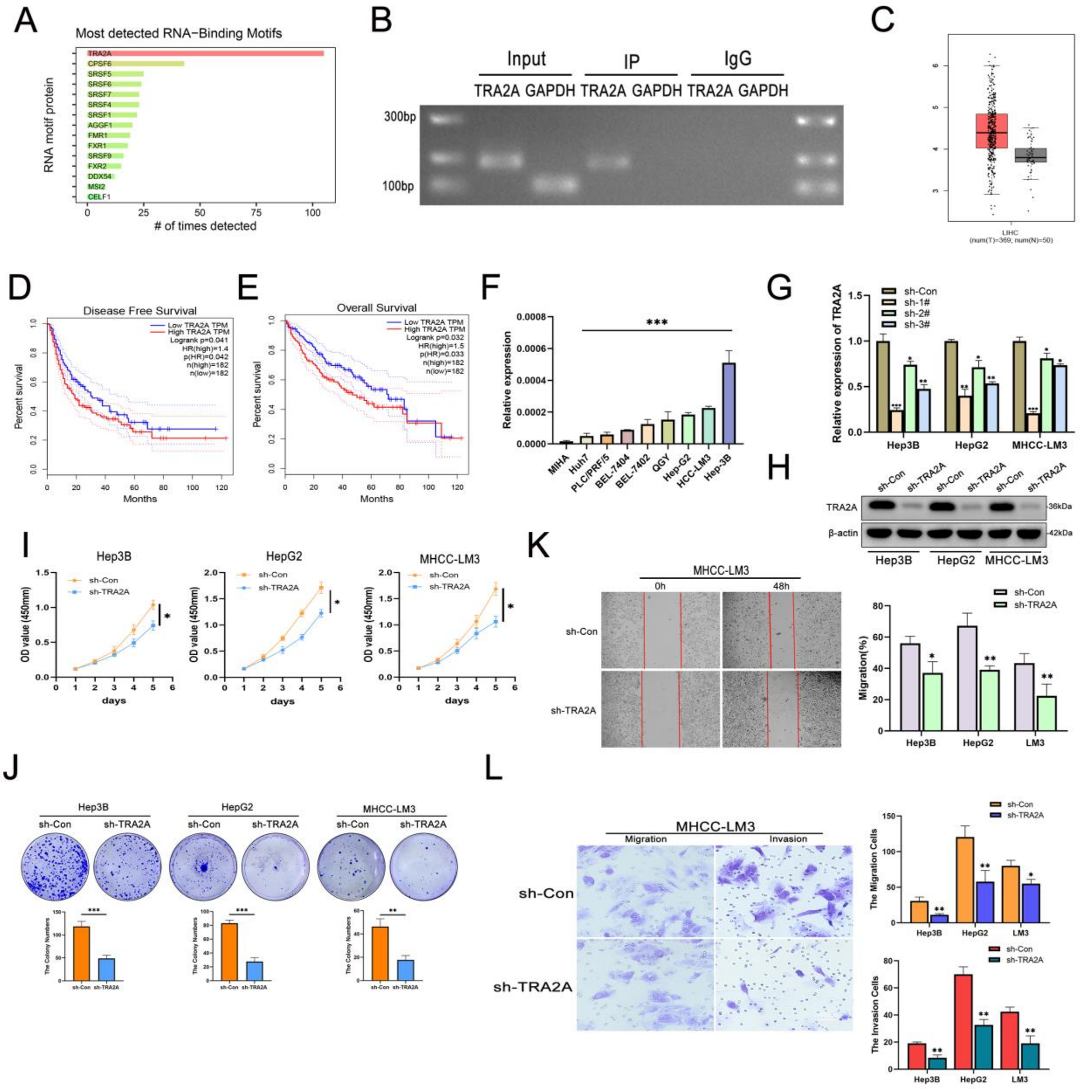

Supplementary figure S3

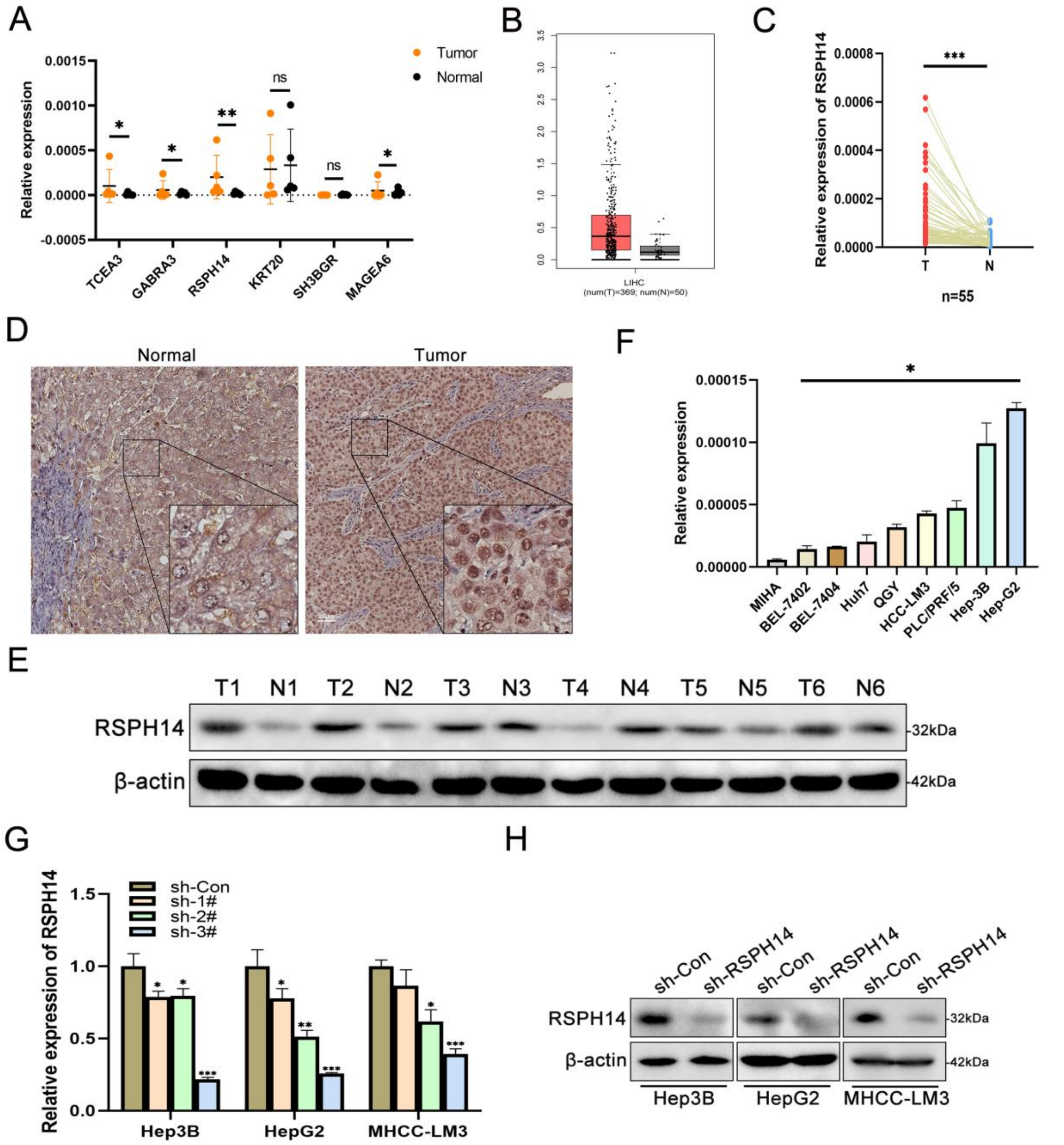

Supplementary figure S4

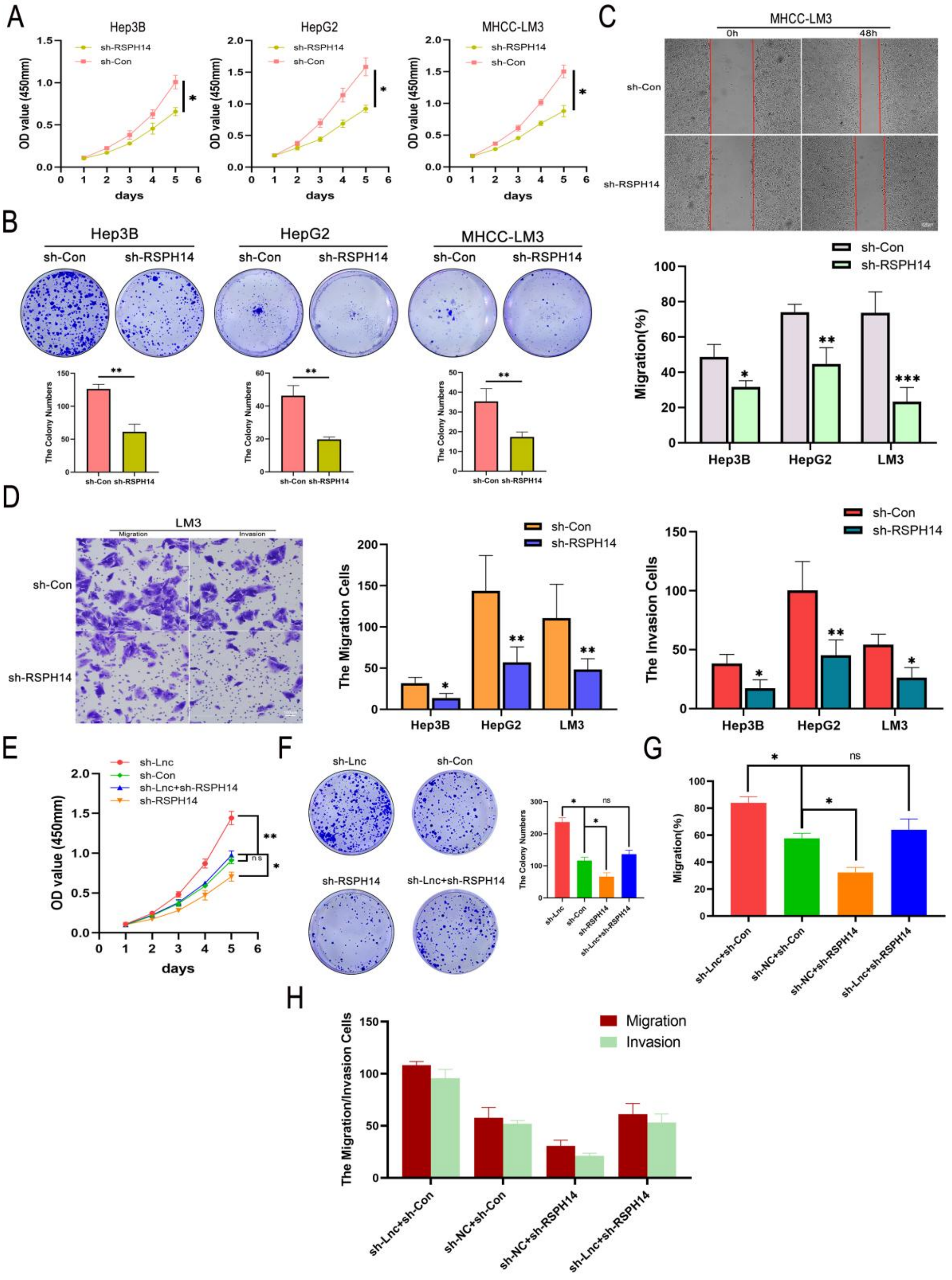

Supplement: Supplementary file 1 — Supplementary figures and tables. [file ijbsv19p3678s1.pdf]
